# Mutations of Cx43 that affect B cell spreading in response to BCR signaling

**DOI:** 10.1242/bio.20147328

**Published:** 2014-02-13

**Authors:** Letitia Falk, May Dang-Lawson, José Luis Vega, Farnaz Pournia, Kate Choi, Caren Jang, Christian C. Naus, Linda Matsuuchi

**Affiliations:** 1 CELL and I-cubed (I^3^) Research Groups, Life Sciences Institute, University of British Columbia, 2350 Health Sciences Mall, Vancouver, BC V6T 1Z3, Canada; 2 Department of Zoology, Life Sciences Institute, University of British Columbia, 2350 Health Sciences Mall, Vancouver, BC V6T 1Z3, Canada; 3 Department of Physiology, Pontificia Universidad de postal codes for Chile are in the Author queries table Católica de Chile, 8330025 Santiago, Chile; 4 Experimental Physiology Laboratory (EPhyL), Instituto Antofagasta, Universidad de Antofagasta, 1270300 Antofagasta, Chile; 5 Department of Cellular and Physiological Sciences, Life Sciences Institute, University of British Columbia, 2350 Health Sciences Mall, Vancouver, BC V6T 1Z3, Canada; 6 Cell and Neuroscience Research Groups, Life Sciences Institute, University of British Columbia, 2350 Health Sciences Mall, Vancouver, BC V6T 1Z3, Canada

**Keywords:** B cell, B cell receptor, BCR, Cx43, Connexin 43, Hemichannel, Gap junction protein

## Abstract

The gap junction (GJ) protein connexin 43 (Cx43) is both necessary and sufficient for B cell receptor (BCR)-mediated cell spreading. To address how Cx43 mediates this effect, we blocked its function genetically, by expressing mutants of Cx43, and pharmacologically, by using chemical inhibitors. While various point mutations of Cx43 inhibited B cell spreading, treatment with channel blocking drugs did not, suggesting that this response was independent of channel function. The critical region of Cx43 appears to be the cytoplasmic carboxyl-terminal (CT) domain, which has previously been shown to be important for B cell spreading. Consistent with this, mutations of either tyrosine 247 or 265 found in the CT were sufficient to inhibit spreading. Thus Cx43 may influence B cell spreading by mechanisms requiring protein binding to, or modification of, these sites in the CT tail.

## INTRODUCTION

Recognition of antigen (Ag) by the BCR leads to B cell proliferation and differentiation. Batista and others have shown that B cells undergo dynamic spreading upon BCR stimulation which facilitates the formation of the immune synapse (Fleire et al., 2006), prolongs BCR signaling and improves Ag internalization, as well as increasing BCR mobility and microcluster-formation (Batista et al., 2001; Carrasco et al., 2004). Stability of the cortical actin cytoskeleton and mobility of BCRs in the plasma membrane depend upon membrane microdomains and the integral membrane proteins adjacent to BCR signaling microclusters. Membrane proteins that affect BCR signaling include the co-receptor CD19 and the tetraspanin CD81 (Mattila et al., 2013). The importance of associated membrane proteins for BCR signaling led us to consider other candidates that might influence BCR signaling and changes in the cytoskeleton.

Using both loss-of-function and gain-of-function strategies, we previously showed the importance of Cx43 for cell spreading in response to BCR signaling (Machtaler et al., 2011). Loss of Cx43 has well documented effects on the cytoskeleton which have been reviewed (Matsuuchi and Naus, 2013). Mutational studies of Cx43 in neural migration have highlighted important domains of Cx43 yet these results are not completely understood. The CT domain of Cx43 is implicated in glioma migration since CT truncation impedes cell migration (Bates et al., 2007). Yet migration is unaffected by treatment with the channel-blocking drug carbenoxolone (CBX), suggesting that migration is not regulated by GJ channel conductance (Bates et al., 2007).

We have previously shown that the CT tail (amino acids 246–382) was necessary for J558µm3 cell spreading in response to BCR-stimulation (Machtaler et al., 2011). How the CT acts in these processes remains to be determined. Truncation of the CT reduces GJ conductivity (Behrens et al., 2010), which might suggest that GJ communication is required for Cx43-influenced processes like BCR-mediated cell spreading; however, spreading B cells in our system were plated at low density such that they do not make cell–cell contacts and form GJs. Increasing evidence that Cx43 forms undocked hemichannels allowing exchange of small molecules between the cell cytoplasm and the extracellular environment suggests that hemichannel activity of Cx43 could influence cell spreading. The function of Cx43 hemichannels has been poorly explored in B cells; however, increasing evidence suggests that hemichannels play a role in communication in the immune system (Junger, 2011; Mendoza-Naranjo et al., 2011; Woehrle et al., 2010). Given the importance of hemichannels in immunity and the high expression of Cx43 by B cells during stages of development when transient contacts with endothelial/stromal cells are made (Orellana et al., 2009), addressing the possible involvement of Cx43 hemichannels in B cell processes was important to explore.

## RESULTS AND DISCUSSION

### Overexpression of Cx43 in the B cell line J558µm3 results in hemichannel activity

**Fig. 1.**
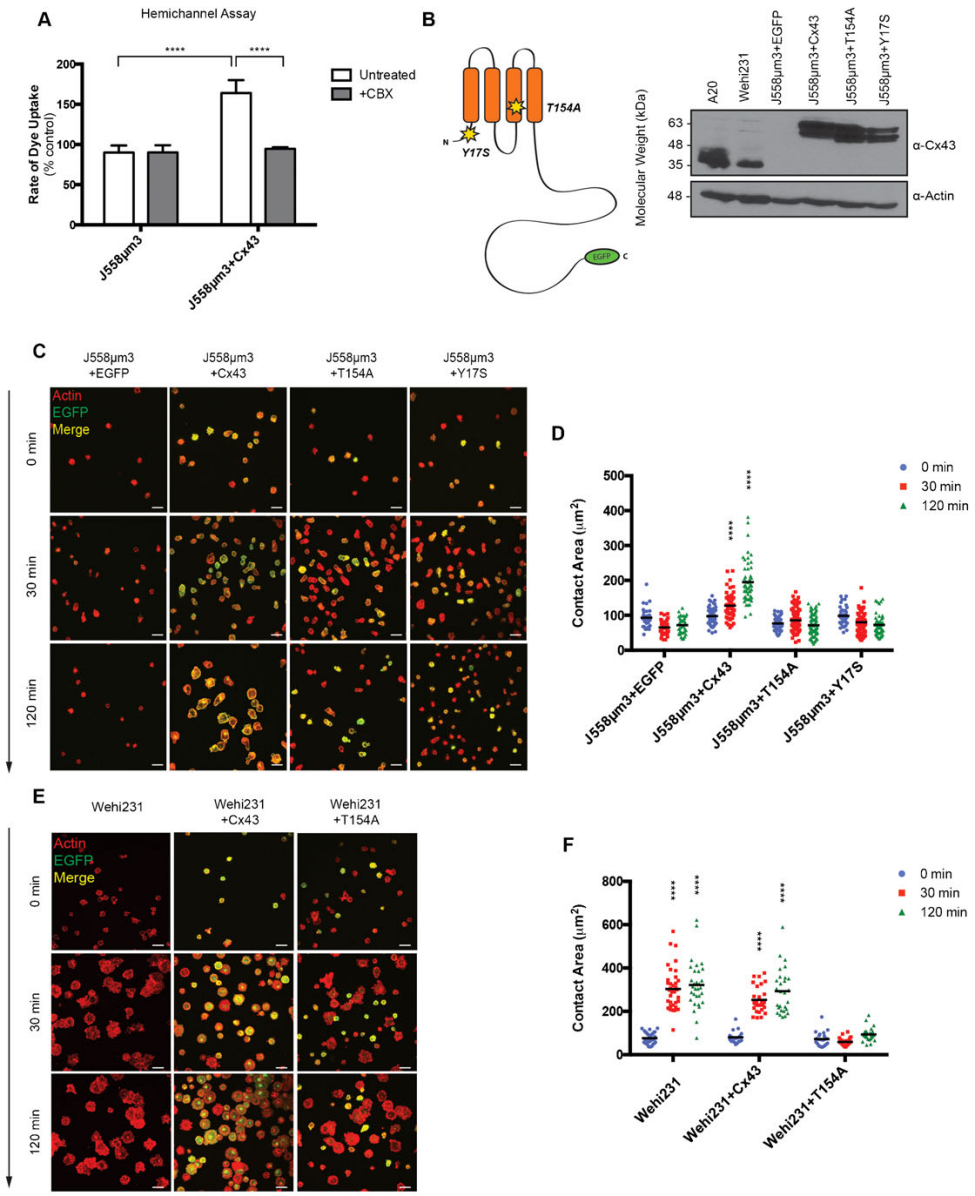
Cx43 mutations T154A and Y17S inhibited radial spreading by J558µm3 and Wehi231 B cells in response to BCR signaling. (A) Hemichannel activity in J558µm3 overexpressing Cx43-EGFP. Rate of dye uptake is shown as a percentage of astrocyte dye uptake in buffer containing divalent cations. ****P<0.0001, *n* = 100. (B) Schematic showing locations of predicted channel-blocking Cx43 mutations T154A and Y17S, location of the EGFP tag, and expression in cell lines by immunoblot. (C) BCR-mediated spreading of J558µm3. (D) Quantification of spreading shown in panel C. Asterix denotes significant difference between later time-points compared to 0 min as determined by P-value: ****<0.0001, *n* = 100. (E) BCR-mediated spreading of Wehi231. (F) Quantification of spreading shown in panel E. Scale bars: 20 µm.

The J558µm3 plasmacytoma B cell line expresses a full 4 chain (IgM, Igλ, Igα, and Igβ) BCR at the plasma membrane and is ideal for the study of Cx43 mutants since it does not express endogenous Cx43, which could otherwise mask the effects of the expression of mutated Cx43 by forming heteromeric hexamers with WT Cx43. The J558 cell line has been used previously to reconstitute and study BCR signaling (Dylke et al., 2007; Flaswinkel and Reth, 1994; Jang et al., 2010; Justement et al., 1990). Since J558µm3 does not endogenously express Cx43, it was not surprising when we were unable to detect hemichannel conductance. In contrast, ectopic expression of Cx43-EGFP was sufficient to drive a significant increase in hemichannel activity that could be blocked by pre-treatment of cells with the channel-blocking drug carbenoxolone (CBX) (Fig. 1A). In order to definitively determine if Cx43 hemichannels influenced B cell spreading, J558µm3 cells were transfected with vectors containing EGFP-tagged Cx43 mutations predicted to block channel activity, and various B cell lines expressing Cx43 were treated with drugs that are known to block GJ channels.

### Cx43 mutants T154A and Y17S inhibit radial spreading in B cell lines

**Fig. 2.**
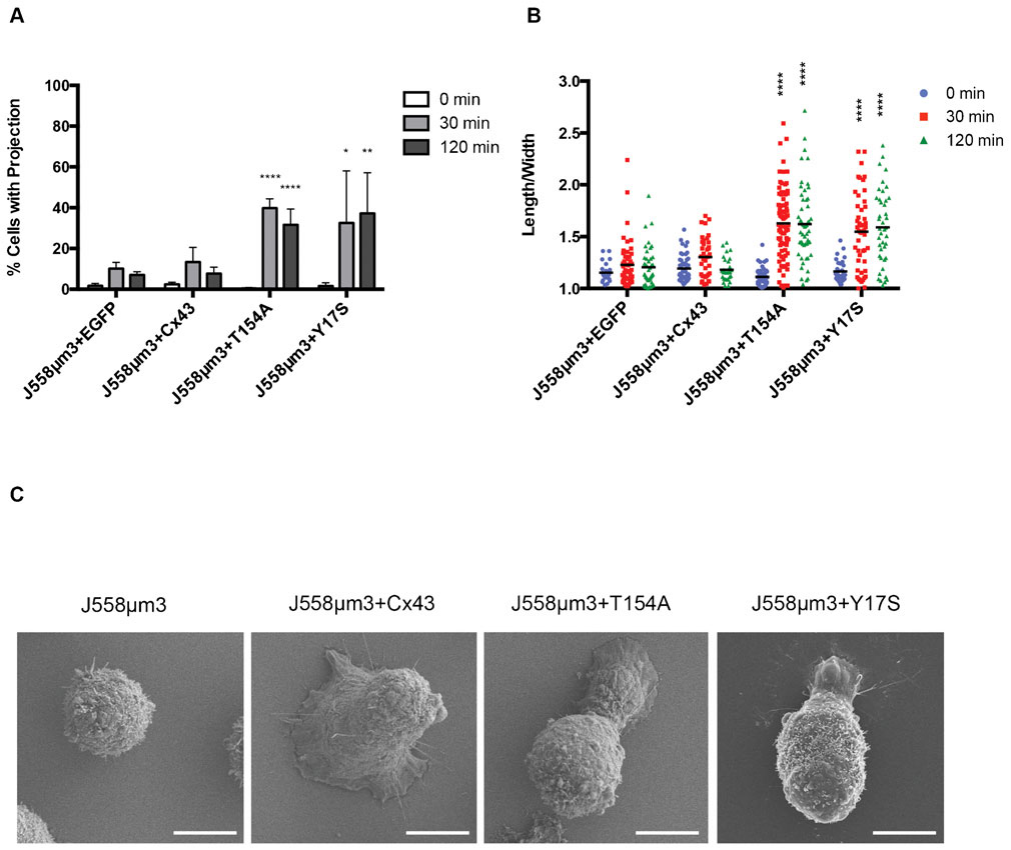
Expression of Cx43 mutant T154A resulted in non-radial spreading and formation of protrusions in J558µm3 cells spreading in response to BCR signaling. (A) Mean percentage of cells with protrusions during BCR-induced cell spreading. Protrusions were defined as cellular extensions with lengths equal to at least half the cell diameter or greater. Asterix denotes significant difference between later time-points compared to 0 min as determined by P-value: *<0.05; **<0.005; ****>0.0001, *n* = 100. (B) Quantification of roundness by length/width where 1  =  round and >1 indicates non-radial spreading and the formation of protrusions. Asterix denotes significant difference between later time-points compared to 0 min as determined by P-value: ****<0.0001, *n* = 100. (C) SEM of spreading J558µm3 cells expressing the given constructs. Scale bars: 5 µm.

Cx43 point mutants T154A and Y17S (Fig. 1B) have both been used as channel-blocking tools (Francis et al., 2011; Good et al., 2011; Zucker et al., 2013). The Cx43 channel mutants were EGFP-tagged and expression was confirmed by immunoblotting (Fig. 1B). Fusion of EGFP to the Cx43 cytoplasmic terminus has previously been determined to not impair trafficking or GJ activity in NRK, MDCK, HeLa, and N2A cells (Jordan et al., 1999; Laird et al., 2001). We have previously confirmed that Cx43-EGFP is expressed at the surface of J558µm3 cells and other lymphoid cells by immunofluorescence and by biotinylation of surface proteins and pull down using avidin-coated beads (Falk, 2013). Cx43 mutant protein localization at the plasma membrane with the BCR was confirmed by confocal microscopy (supplementary material Fig. S1A). Ectopic overexpression of Cx43 (WT or mutated) did not influence cell size or proliferation (supplementary material Fig. S1B,C). Unlike WT, expression of neither T154A nor Y17S was sufficient to cause J558µm3 spreading (Fig. 1C, quantified in Fig. 1D). However, approximately half of the cells expressing channel mutations formed asymmetric protrusions in response to BCR signaling, as compared to less than 20% of the cells expressing WT Cx43 or EGFP alone (Fig. 2A). The length of these protrusions varied from half the cell diameter to twice its length and extended in sporadic directions (Fig. 2B,C). These types of protrusions are characteristic of mobile B lymphocytes (Anderson et al., 1979) and not a defect caused by overexpression.

T154A and analogous mutations of conserved threonine residues in other Cxs (i.e. T135 and T157 found in Cx26 and Cx50, respectively), act as dominant negatives on GJ permeability and on non-channel functions such as migration and growth suppression (Beahm et al., 2006; Good et al., 2011; Zucker et al., 2013). Expression of T154A in the Cx43-expressing immature B cell line Wehi231 inhibited cell spreading, in contrast to overexpression of WT Cx43 and to normal, untransfected Wehi231, both of which spread significantly on anti-IgM coated coverslips (Fig. 1E, quantified in Fig. 1F). In summary, the GJ channel-blocking Cx43 point mutations T154A and Y17S inhibited BCR-mediated radial spreading in B cells, but unlike the ΔCT mutant, these transfected cells retained the ability to rearrange their cytoskeleton enough to form asymmetric protrusions characteristic of motile B cells.

### Hemichannel activity is not required for B cell spreading

**Fig. 3.**
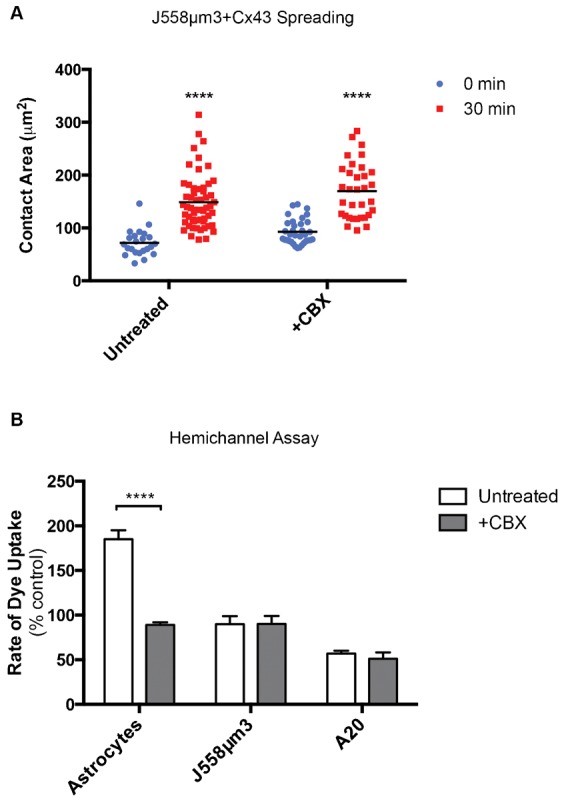
Hemichannel activity is not required for B-cell spreading. (A) BCR-mediated spreading. Asterix denotes significant difference between later time-points compared to 0 min as determined by P-value ****<0.0001, *n* = 50 (no significant difference between treatments). (B) Hemichannel activity of different B cell lines and primary astrocytes in buffer without divalent cations. Significant difference between untreated and CBX-treated cells ****P<0.0001, *n* = 50.

We next tested the effect of the channel-blocking drug, CBX, on cell spreading. Cx43 expressing J558µm3 cells were treated with 100 µM CBX, which blocked Cx43 hemichannel activity (Fig. 1A). In contrast to the channel-blocking point mutations, blocking hemichannels with CBX did not inhibit cell spreading (Fig. 3A). CBX treatment also failed to inhibit spreading of three types of Cx43-positive B cells, Wehi231, A20, and primary splenic B cells. Treatment with channel blocking drugs, probenecid (Pbn, 1 mM, blocks pannexin channels) and lanthanum (La^3+^, 200 µM, blocks Cx channels) also had no effect on B cell spreading (supplementary material Fig. S2A–D).

The mature B lymphoma cell line A20 expresses high levels of Cx43 (Fig. 1C), allowing us to ask whether endogenous levels of Cx43 were sufficient for hemichannel activity. Unlike J558µm3 cells overexpressing Cx43, A20 cells did not have hemichannel activity (Fig. 3B). Taken together, these data suggest that hemichannel conductance is not a requirement for BCR-mediated B cell spreading. This sharply contrasts with our observations that ectopic expression of point mutations expected to form non-functional Cx43 GJ channels does hinder BCR mediated B cell spreading in J558µm3 cells (Figs 1 and 2). We reasoned that if channel-activity has no effect on cell spreading, then an alternative possibility is that T154A and Y17S mutations cause a secondary defect in the Cx43 molecule other than the well-known channel disruption phenotype and this secondary defect may have contributed to the abrogated cell spreading. For example, analogous mutations in other Cxs generate oligomers that are less stable, or form hexamers that do not pack as tightly into GJ plaques (Beahm et al., 2006). In particular, we hypothesized the T154A mutation could influence the conformation of the CT and thus availability of important protein interaction sites found within this region (Ponsaerts et al., 2012), leading us to next look at the CT tail.

### Defined regions of the Cx43 CT domain are required for B cell spreading

**Fig. 4.**
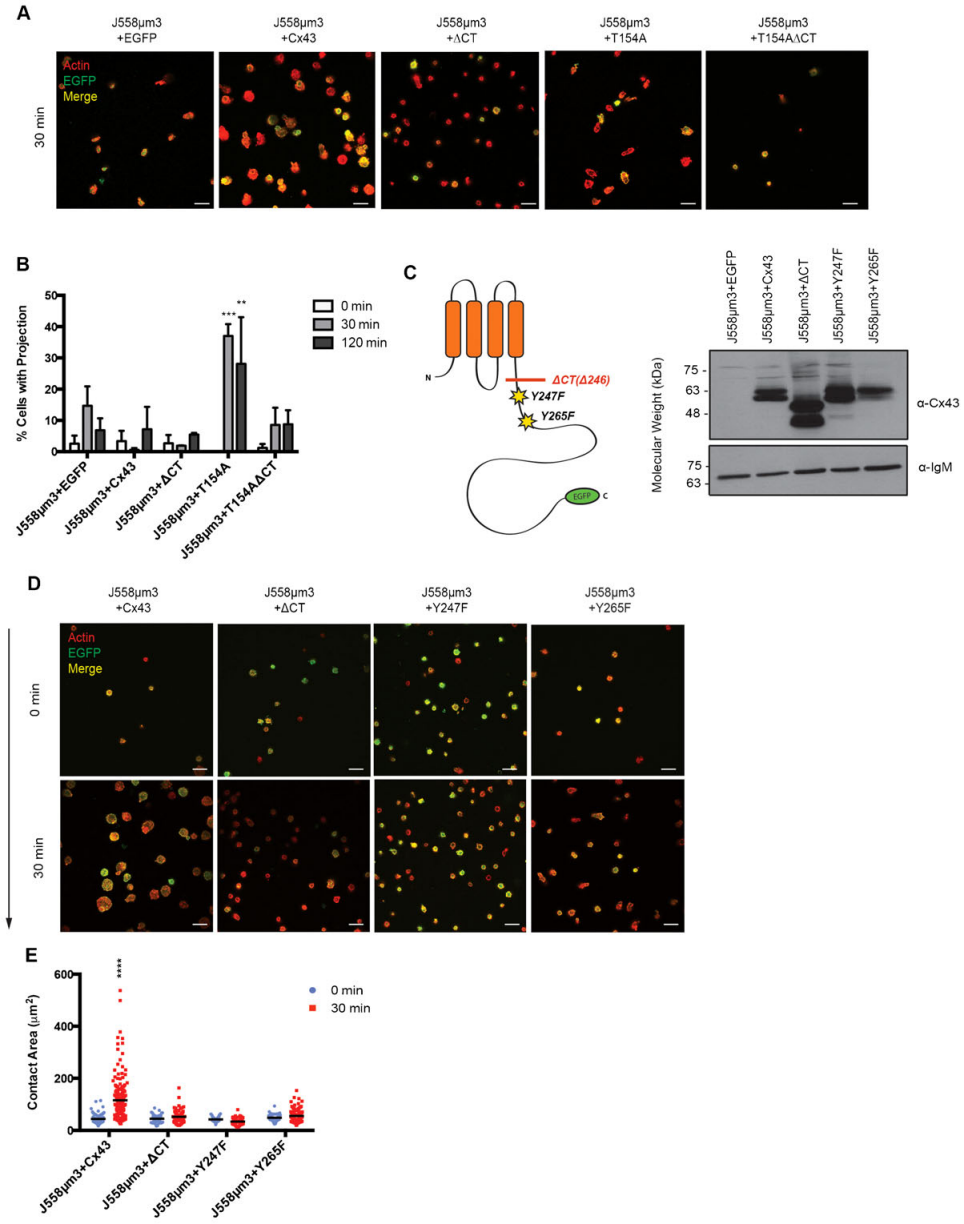
The importance of the Cx43 CT domain for B cell spreading in response to BCR signaling. (A) BCR-mediated spreading. (B) Mean percentage of cells exhibiting non-radial spreading. Asterix denotes significant difference between later time-points compared to 0 min as determined by P-value: **<0.005; ***<0.001, *n* = 100. (C) Schematic showing the locations of EGFP-tagged Cx43 mutants in the cytoplasmic tail. Expression of the transfected Cx43 forms by J558µm3 cell lines shown by immunoblot. (D) BCR-mediated spreading. (E) Quantification of spreading shown in panel D. Asterix denotes significant difference between later time-points compared to 0 min as determined by P-value: ****<0.0001, *n* = 100. Scale bars: 20 µm.

Expression of Cx43 mutants T154A or Y17S resulted in the formation of protrusions which could represent an intermediate stage which would normally lead to radial spreading. In order to determine the importance of the CT in this response, a carboxyl-terminal truncation at amino acid 246 was generated in the Cx43 mutant T154A (T154AΔCT) to determine whether loss of the CT tail could override the formation of protrusions caused by mutation of T154. In response to BCR signaling, J558µm3+T154AΔCT neither spread radially nor formed protrusions (Fig. 4A,B). This suggests that T154A inhibits cell spreading independently of channel activity, possibly by affecting function of the CT, which we predict acts as a site for protein interactions linking Cx43 to events downstream of BCR signaling.

The CT region of Cx43 was also required for activation of the Rap1 GTPase downstream of BCR signaling (Machtaler et al., 2011). Rap1 is required for cytoskeletal rearrangements leading to B cell spreading (Lin et al., 2008; Machtaler et al., 2011; McLeod et al., 1998). To confirm that Cx43 expression influences spreading through Rap activation, J558µm3 cells stably expressing Cx43-EGFP were transiently transfected with the dominant negative, FLAG-tagged RapGAPII which converts Rap1 into its inactive, GDP-bound form (McLeod et al., 1998; McLeod et al., 2002). FLAG-tagged RapGAPII positive cells were stained with anti-FLAG and did not spread compared to untransfected J558µm3+Cx43 cells, indicating that the effect of Cx43 on spreading depends on Rap1 activation (supplementary material Fig. S3A, quantified in supplementary material Fig. S3B). Consistent with this finding, expression of a FLAG-tagged, constitutively active Rap1V12 (McLeod et al., 2002) caused J558µm3 cells, which normally do not spread, to spread radially (supplementary material Fig. S3A, quantified in Fig. S3B). Unlike its effect on spreading, expression of T154A had minimal effects on the activation of Rap1 in response to BCR signaling (supplementary material Fig. S3C), even though these transfected cell lines had equivalent levels of membrane IgM (supplementary material Fig. S3D), ensuring equal stimulation through the BCR. One caveat is that J558 plasmacytoma cells do not express CD19 (data not shown). However, knockdown of Cx43 also reduced spreading in the CD19 positive Wehi231 cell line (Machtaler et al., 2011), indicating that Cx43 can influence BCR-mediated spreading independently of CD19. Thus our results may reflect a more general influence of Cx43 on B cell processes that require Rap1 and cytoskeletal remodeling since Cx43 is also required for integrin and chemokine dependent Rap1 activation and for B cell motility and migration (Machtaler et al., 2011).

To extend these findings, EGFP-tagged mutations of two tyrosines in the CT tail, Y247 and Y265 with proposed roles in Src kinase binding (Montecino-Rodriguez et al., 2000), were made, and their expression in J558µm3 cells were characterized by immunoblotting (Fig. 4C) and protein localization determined by confocal microscopy (supplementary material Fig. S1D). J558µm3 cells expressing either Y247F or Y265F did not spread in response to BCR signaling (Fig. 4D, quantified in Fig. 4E). This shows that tyrosine residues 247 and 265 of the Cx43 tail are necessary for B cell spreading. It remains to be determined how these tyrosine residues influence B cell spreading, therefore future studies will explore if Y247 and Y265 are required for the normal structure and topology of the Cx43 protein and CT tail, or if they act as binding sites for interacting proteins such as those found in GJ proteome studies (Laird, 2010).

## MATERIALS AND METHODS

### Plasmids and cell lines

The NAP2 expression vector containing WT Cx43 with EGFP fused in-frame to the CT tail and ΔCT, as well as the AP2 vector which contains EGFP alone have been described previously (Bates et al., 2007). The Cx43 mutant T154A was generated using previously published primers (Beahm et al., 2006). Site-directed mutagenesis was performed on WT Cx43 and ΔCT, to generate the T154A mutation in these expression vectors. The J558µm3 plasmacytoma B cell line which expresses a full 4 chain (IgM, Igλ, Igα and Igβ) BCR at the plasma membrane was a gift from Dr Louis Justement (University of Alabama, Birmingham) (Justement et al., 1990). The BOSC 23 retroviral packaging cell line was a gift from Dr Warren S Pear (Massachusetts Institute of Technology, Cambridge) (Pear et al., 1993). Wehi231 and A20 B cell lines were obtained from the American Type Culture Collection (Rockville, Maryland). Primary murine B cells were isolated according to the manufacturer's instructions from spleens of C57Black6 mice using the EasySep® mouse B cell enrichment kit from Stem Cell Technologies. Proliferation was measured by Alamar Blue (Sigma) reduction, measured using a fluorescent plate reader.

### Retroviral transduction

J558µm3 or Wehi231 cells were transduced with 2 µg of mutated or WT EGFP-fused Cx43 plasmid DNA using the retroviral packaging cell line, BOSC 23 via calcium phosphate precipitation (Krebs et al., 1999). Viral supernatant was collected at 24, 48, and 72 h post-transduction, and used to infect 0.5×10^6^ J558µm3 or Wehi231 cells. Stably transduced B cells were sorting for high-EGFP by fluorescence-activated cell sorting (FACS), with the assistance of the UBC FACS Facility.

### Preparation of cell extracts and antibodies

Cell lysis was performed as described (Machtaler et al., 2011). For immunoblotting, goat α-mouse IgM (μ chain specific) was from Jackson Immuno Research Laboratories. Rabbit α-mouse antibody against the CT tail of Cx43 (amino acids 363–382) was from Sigma, whereas the mouse α-mouse antibody against the N-terminal region of Cx43 (amino acids 1–20) was from the Fred Hutchinson Cancer Research Institute. Rabbit α-mouse Rap1 was from Cell Signaling Technologies. Mouse α-mouse actin was from Fisher Scientific.

### Pharmacological blocking of hemichannels and assay of hemichannel activity

Cells were incubated 30 min at 37°C with one of: 100 µM CBX (Sigma), 1 mM Pbn (Alfa Aesar), or 200 µM La^3+^ (Sigma). Blocked cells were either added directly to anti-IgM coated coverslips for spreading assay or used in hemichannel assay. Hemichannel activity was measured using a procedure adapted from Orellana et al. (Orellana et al., 2009). For time-lapse fluorescence imaging, 5×10^6^ cells washed and resuspended in Locke's solution or with or without divalent cations to induce opening of hemichannels, containing 5 µM ethidium bromide (EtBr). Fluorescence intensities were measured using Axiovision software with images acquired by a Zeiss Axioplan2 epifluorescence microscope. Images were collected every 30 sec for 15 min and analyzed with the ImageJ program (NIH software). The rate of dye uptake was used to measure hemichannel activity and was quantified by calculating the slope of the mean fluorescence intensity (MFI)/time (s). Rate of dye uptake was displayed as a percentage of the mean dye uptake by astrocytes measured in regular Locke's buffer.

### In vitro cell spreading assay

Cell spreading assays were performed as described (Lin et al., 2008; Machtaler et al., 2011). To quantify spreading, contact area (µm^2^) at the cell–coverslip interface was measured using stained actin to locate the cell periphery. Images were taken on an Olympus Fluoview 1000 confocal microscope and Image Pro Plus 6.2.

### Scanning electron microscopy

Samples were prepared for SEM using standard procedures at the UBC Bioimaging Facility.

### Cell stimulation and Rap activation assay

Rap activation was performed as has been described (McLeod et al., 1998). Briefly, cells were stimulated with anti-IgM and lysed. The Rap1 binding domain of the Ral-GDS protein was used to pull down only the active, GTP-bound form of Rap1.

### Statistical analysis

A 2-way analysis of variance (ANOVA) calculated by Prism Graph Pad, was used to determine the significance of time and expression on contact area, percentage cells with projections, and length/width, and student's two-tailed, unpaired t-tests calculated by Prism Graph Pad or Microsoft Excel were used to do pair-wise comparison of means of contact area, % cells with projections, length/width, and dye-uptake. Asterisks represent significance based on a 95% confidence interval (P<0.05). For spreading assays, asterisks represent comparisons between later time-points and the 0 min control. Error bars on graphs show standard error of the mean.

## Supplementary Material

Supplementary Material
