# Supplementary material for: Mutations of Cx43 that affect B cell spreading in response to BCR signaling
Source: Biol Open. 2014 Feb 13;3(3):185–94. doi: 10.1242/bio.20147328 (PMC4001238; doi:10.1242/bio.20147328)
Supplement: Supplementary Material [file supp_3_3_185__index.html]

Mutations of Cx43 that affect B cell spreading in response to BCR signaling — Mutations of Cx43 that affect B cell spreading in response to BCR signaling — Supplementary Material 

# Mutations of Cx43 that affect B cell spreading in response to BCR signaling

## bio.20147328 Supplementary Material

**Files in this Data Supplement:**

- Supplementary Material - Letitia Falk et al. doi: 10.1242/bio.20147328
