# Supplementary material for: Mutations of Cx43 that affect B cell spreading in response to BCR signaling
Source: Biol Open. 2014 Feb 13;3(3):185–94. doi: 10.1242/bio.20147328 (PMC4001238; doi:10.1242/bio.20147328)
Supplement: Supplementary Material [file supp_bio.20147328_bio.20147328-s1.pdf]

 Biology Open (2014) 000, 1–7 doi:10.1242/bio.20147328

Supplementary Material
Letitia Falk et al. doi: 10.1242/bio.20147328

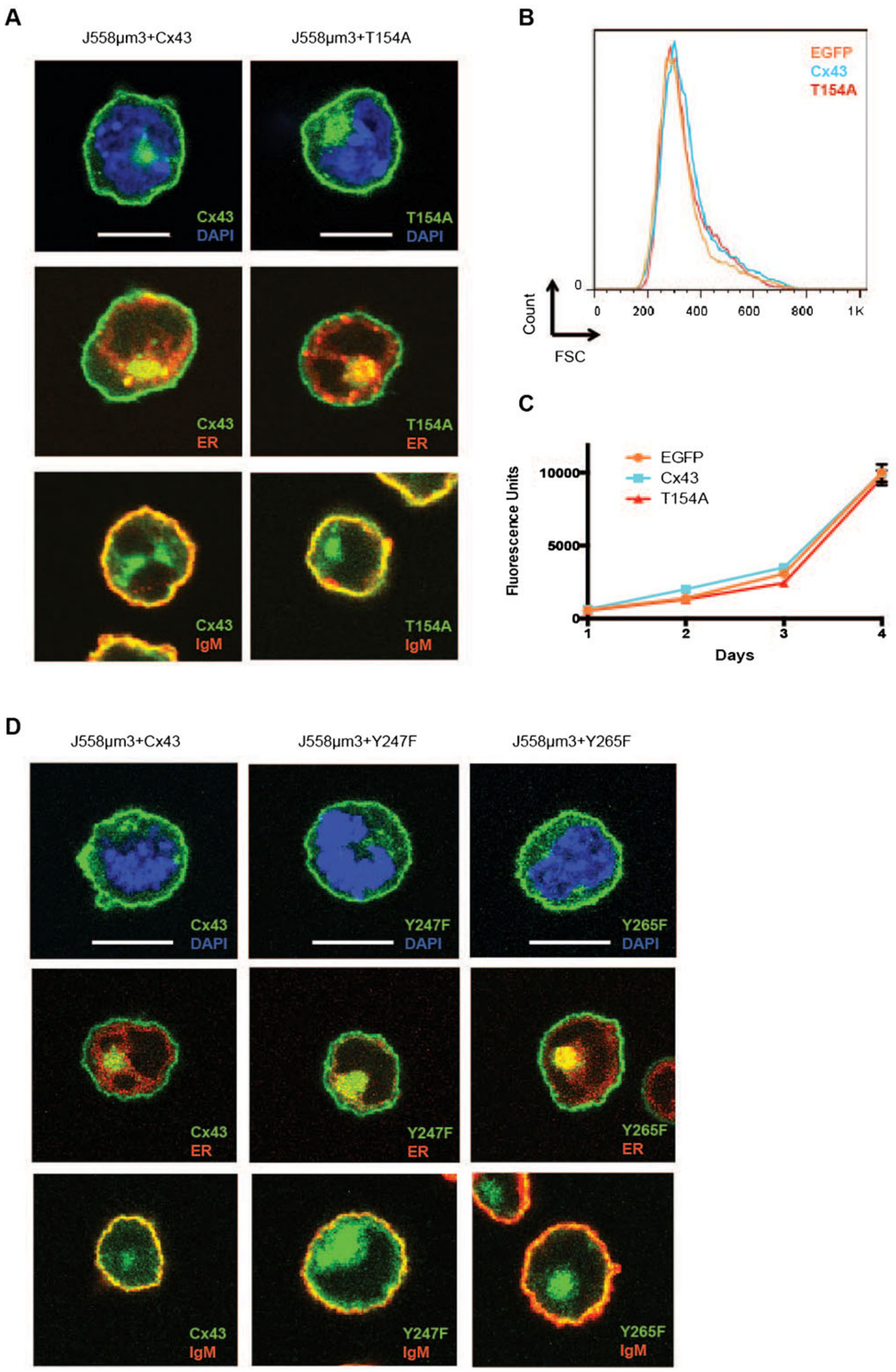

Fig. S1. Characterization of J558μm3 cells expressing Cx43 mutations. (A) Localization of EGFP-fused WT or T154A Cx43 expressed by J558μm3 cells. (B) Cell size of indicated transfected cells, measured by forward scatter (FSC) using flow cytometry. (C) Cx43 and T154A expression did not affect cell proliferation over a 4-day period. Error bars represent standard error of the mean. Data are representative of three separate experiments, each done in triplicate. (D) Localization of EGFP-fused Y247F or Y265F Cx43 expressed by J558μm3 cells as described in panel A. Scale bars: 10 μm.

Biology Open

Biology Open (2014) 000, 1–7 doi:10.1242/bio.20147328

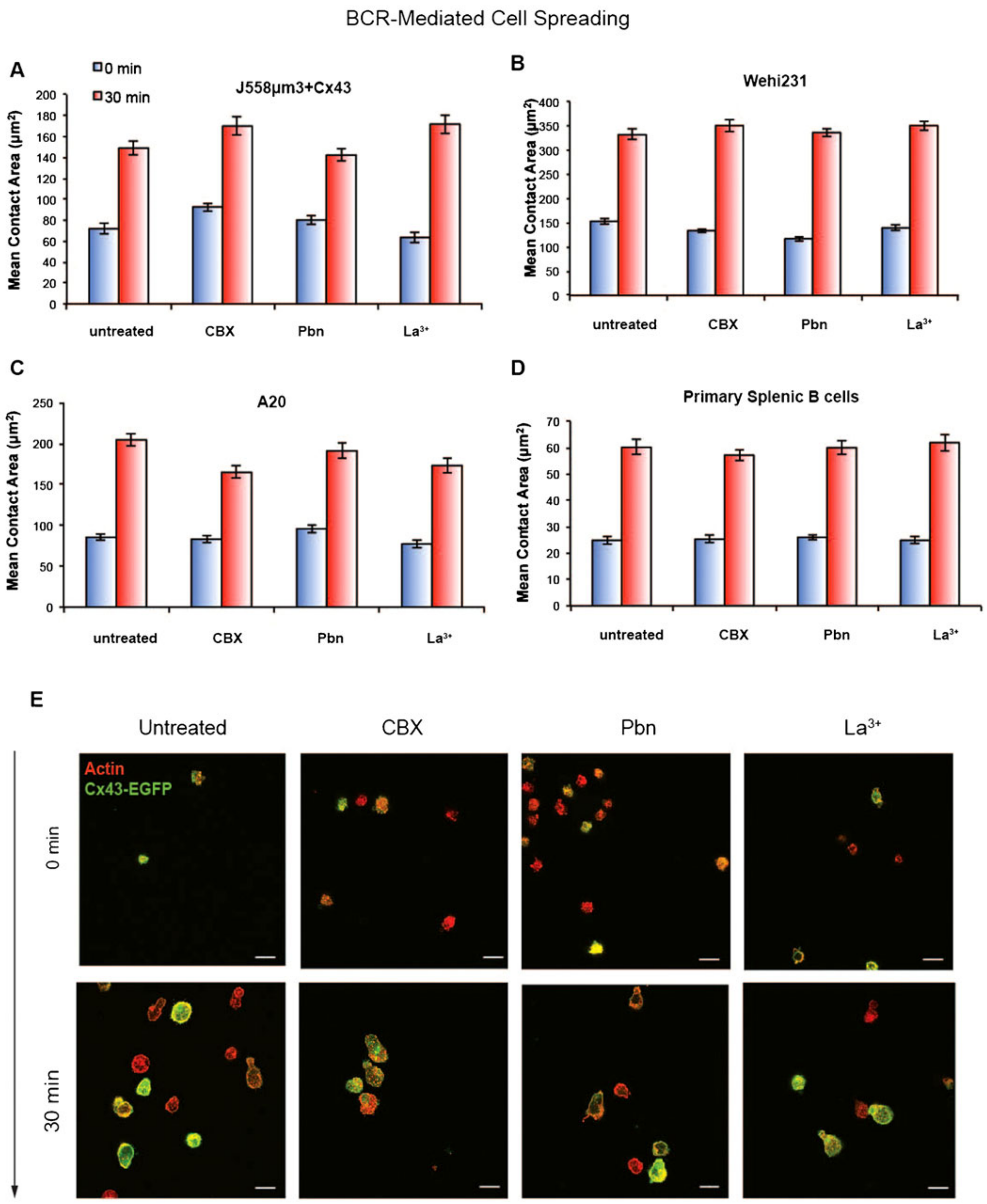

Fig. S2. Effect of channel blocking drugs on spreading of different types of B-lymphocytes in response to BCR signaling. Quantification of contact areas as a measure of BCR-mediated cell spreading of (A) J558µm3 cells expressing transfected Cx43-EGFP, (B) Wehi231 B lymphoma cells, (C) primary splenic B-lymphocytes, and (D) A20 B lymphoma cells. Cells were pre-treated with channel blocking drugs (100 µM CBX, 1 mM Pbn, 200 µM La^{3+}), or left untreated as a control. No significant difference between treatment types. (E) Images of spreading J558µm3 overexpressing WT Cx43-EGFP. Scale bars: 10 µm.

Biology Open

Biology Open (2014) 000, 1–7 doi:10.1242/bio.20147328

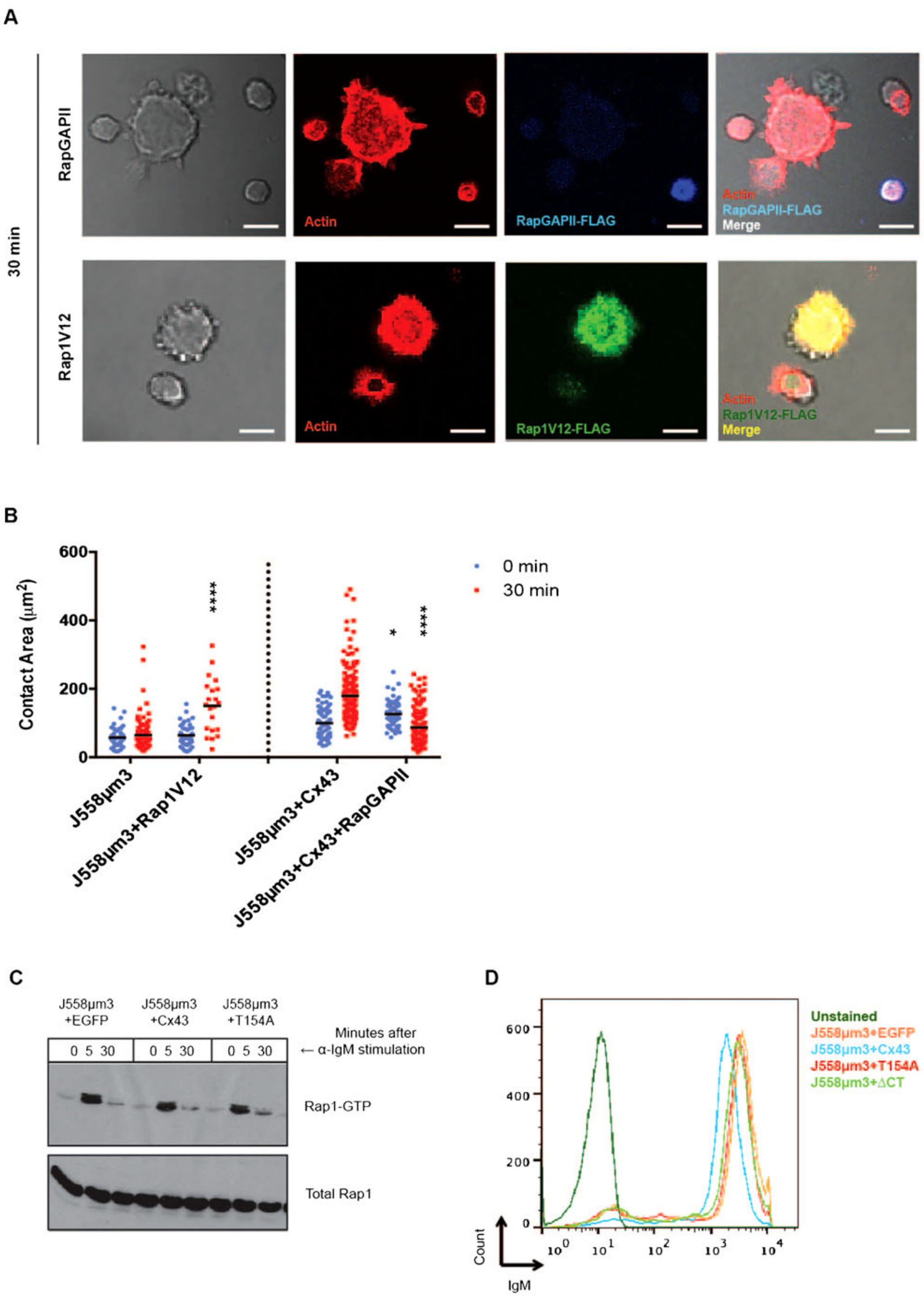

Fig. S3. The influence of Cx43 on BCR-mediated spreading of J558μm3 cells depends upon Rap1 activation. (A) BCR-mediated cell spreading of J558μm3 cells stably expressing Cx43-EGFP transiently transfected with RapGAPII-FLAG (upper panel), or J558μm3 transiently transfected with Rap1V12-FLAG (lower panel). Scale bars: 10 μm. (B) Quantification of spreading area as a measure of spreading. Significance between cell types *$P<0.05$; ****$P<0.0001$, $n=50$. (C) Rap1 activation in J558μm3 cells expressing EGFP, or EGFP-fused WT or T154A Cx43. Cells were stimulated for the indicated times with 20 μg/ml of soluble anti-IgM and activated Rap1 was precipitated using a GST-RalGDS fusion protein and total Rap1 detected by blotting with anti-Rap1. (D) Surface BCR was stained using a PE-conjugated anti-IgM antibody and levels were measured by flow cytometry.

Biology Open
